# Correcting “insertion‐deletion mutations” in medical terminology

**DOI:** 10.1111/jcmm.13875

**Published:** 2018-09-06

**Authors:** Alexios‐Fotios A. Mentis, Athanasios G. Papavassiliou

**Affiliations:** ^1^ Public Health Laboratories Hellenic Pasteur Institute Athens Greece; ^2^ Department of Biological Chemistry Medical School National and Kapodistrian University of Athens Athens Greece

The human genome and written language share analogous substructures: linear strings of letters forming functional‐structural molecular units resemble alphabetical letters which, arranged in a linear fashion, form words; these strings become more than the sum of their parts in producing genes or phrases. In biomedicine, key findings obtained from computer science approaches, using linguistic principles within algorithms to search for base letters in DNA sequences—the exponential *bioinformatics* arena—have led to scientific breakthroughs.

An analogous yet a less common perspective reveals that DNA mutations—point and insertion‐deletion mutations (“indels”)—harbour analogies to word evolution, including changes in biomedical terminology. Precision medicine approaches diagnose such DNA mutations by comparing reference genomes with those of clinical specimens to enable personalized therapy.^1^ Similarly, spell checkers correctly align defined and spelled words from a dictionary to those screened in the text.

Could ascertaining the proper naming and spelling to standardize biomedical terminology be as crucial as maintaining genomic DNA integrity? Alterations, in both cases, may be positive or negative at the functional level; however, they still form life's evolution, natural and socio‐linguistic, respectively. Besides, DNA is much more the master of our bodies’ cellular life and history, and language evolves to meet the zeitgeist, while correctly named medical terminology may be a necessary long‐term endeavour.

For example, the current spelling of leukaemia (in English) is the result of a linguistic mutation. Virchow joined the Ancient Greek words for white (leukos) and blood (haima) to form “Leukämie.” However, leukaemia was misspelled from its inception. During the Hellenistic period, polytonic diacritics incorporated pitch accents to facilitate learning Greek. Words like “αίμα” (blood) were punctuated with (‘), indicating rough breathing and corresponding to a /h/ sound before the first letter (i.e. “haima”). Yet, /ai/ in Greek corresponds to /ae/ in English (“haema”). To produce eloquent composite words harbouring consonants at the end of the first word (e.g. /k/ in leuko‐), the last letter yielded its place to /h/ in the beginning of the second word (“haima”).^2^ Thus, the most accurate spelling for this disorder is “Leuhaemia”. However, a spelling indel arose—deleting /ha/ and inserting /k/—when joining the two nouns to form leukaemia.

This example makes the tenuous history of other “suffering” biomedical terms less digestible, collectively adding to the consistent calls‐to‐action for the medical community's undiluted, widespread efforts for standardization. In the burst of biomedical discoveries, how we chart the trajectory of terminology to accurately describe disease is another area requiring standardization.

Little, if any, influence did the name “syphilis” with long lists of attributed names exercise to the medico‐diplomatic bodies of the era to standardize naming schemes. This could be linked to sequential alterations of medical terms influenced by socio‐political perspectives (e.g. the Spanish flu epidemic of 1917‐1918 reflected Spain's neutral stance during World War I and not the origin of the disease pandemic^3^) or by initial geographical descriptions (e.g. *Brucella melitensis* from the first strains isolated in Malta).

Historically, terms have undergone changes from radically different patho(bio)logical understanding or medical errors (e.g. confusion in differentiating between distinct anatomic sites critical for medico‐surgical intervention). Moreover, medical terminology may misguide treatment. Reflex sympathetic dystrophy is a disease named during the American Civil War, probably encompassing post‐traumatic stress disorder. As invasive sympathectomies were not therapeutically superior and the disease may present following minor civilian accidents, the condition was renamed complex regional pain syndrome to provide physicians with alternative treatment options.^4^

Such changes may also reflect social and personal identity stigma (e.g. castration‐resistant prostate cancer)^5^ or misconceptions. In pneumonia, the common English equivalent of aegophony (aeg stands for αίξ—goat) characterizes the auscultated wavering lung sound of a goat (egophony), literally meaning “hearing my inner voice” (ego = myself).

Furthermore, in lieu of schizophrenia, the term *psychosis susceptibility syndrome* may indicate the spectral character of nosological manifestations.^6^ Should personal perception (e.g. patient narratives) suggest disease and skew the opinion of the physician diagnosing the disease in this phenotypic continuum?

Considering the surge of updated medical approaches including (a) precision medicine and sub‐clustering of patients; (b) global health surveillance for the ready‐to‐emerge next pandemic (“Disease X”); (c) medical tourism, as patients seek medical care among global healthcare systems and (d) genomic medicine heralding solutions to diagnostic odysseys while urging novel names to newly discovered diseases, the medical community should prepare for an official nomenclature. Patients ought to be informed and their speech embraced by physicians, so those affected by a disease extending beyond diagnostic classifications receive appropriate medical treatment. Multiple names will only add confusion.

Based on the availability of electronic medical records, online media, and social media as promising cornucopia of health‐related information, emerging trends appear: (a) *“deep phenotyping”* that will identify phenotypes with *“logically constructed hierarchy of phenotypic terms”* based on patient searches and queries^7^ to understand genetic disorders; and (b) *“digital phenotyping”* for diseases defined as *“operationalization by patterns of search activity gathered during engagement with web search engines”*.^8^ However, these trends necessitate a coalition between medical and laymen terminologies^7^ while maintaining the standards of accurate clinical diagnosis.

These examples highlight the need for uniform spelling and naming of Medical Subject Headings (MeSH). Such an impetus is crucial to (i) appropriately integrating scientific neologisms to express jargon (e.g. although RNA analysis should reach clinical prime time for functional description of tumour heterogeneity, it is described using different names— “RNA‐Seq,” “RNAseq,” and “RNA seq”– precluding easy search of the literature)^9^; and (ii) conducting systematic reviews and meta‐analyses requiring concrete search strings or assisted by machine learning techniques.^10^ In the latter case, performing computer‐based automated MeSH indexing and text mining is crucial. Therefore, uniform spelling and naming of MeSH should be the first step towards standardization, in alignment with the World Health Organization's efforts. Such efforts may be didactic to avoid medical and health communication crises with potential cross‐border effects.

Overall, a uniform approach to medical terminology is essential in the current surge of neologisms to ensure the integrity of biomedical literature.

## CONFLICT OF INTEREST

The authors confirm that there are no conflicts of interest.
